# The relationship of pre-corneal to pre-contact lens non-invasive tear breakup time

**DOI:** 10.1371/journal.pone.0247877

**Published:** 2021-06-28

**Authors:** Andrew D. Graham, Meng C. Lin

**Affiliations:** 1 Clinical Research Center, School of Optometry, University of California, Berkeley, California, United States of America; 2 Vision Science Group, University of California, Berkeley, California, United States of America; University of New South Wales, AUSTRALIA

## Abstract

**Purpose:**

To examine the relationship between pre-corneal and pre-contact lens tear film stability (TFS), and to determine whether pre-corneal TFS is a reliable predictor of subsequent pre-lens TFS after a contact lens is placed on the eye.

**Methods:**

667 records met inclusion criteria and were extracted from a soft contact lens multi-study database. Multivariable linear mixed effects models were fit to examine the association between pre-corneal and pre-lens TFS, adjusting for potential confounders and accounting for repeated measures. Receiver Operating Characteristic (ROC) analysis was employed to assess the predictive performance of pre-corneal TFS for subsequent pre-lens TFS. TFS was quantified for this analysis as the non-invasive tear breakup time (NITBUT).

**Results:**

Pre-corneal NITBUT was significantly related to the pre-lens NITBUT at both 10 min (p<0.001) and 2–6 hrs (p<0.001) post-lens insertion. However, the sensitivities of pre-corneal NITBUT for predicting symptom-associated thresholds of pre-lens NITBUT ranged from 50–65%, and specificities ranged from 57–72%, suggesting poor-to-moderate diagnostic performance.

**Conclusions:**

Despite the association of pre-corneal and pre-lens TFS, the inherent lability and sensitivity to environmental exposures of the tear film introduce significant variability into NITBUT measurements. Using pre-corneal NITBUT to identify likely successful contact lens candidates prior to fitting is thus not sufficiently accurate to be relied upon in the clinical setting.

## Introduction

Despite continual technical advances and improvements in soft contact lens wear, symptoms of dryness and discomfort, often leading to patient discontinuation, continue to be of substantial concern [1–4]. When a contact lens is placed on the eye, the pre-corneal tear film is compartmentalized into pre-lens and post-lens segments, each thinner than the original pre-corneal tear film. While perfect agreement has not been established, due primarily to differences in measurement techniques, the most widely accepted estimates of pre-corneal tear film thickness range from 3 to 11 μm [5–11]. Estimates of the thickness of the compartmentalized tear film segments with a soft contact lens *in situ* range from 2 to 7 μm pre-lens [5, 9, 11–13], and from 3 to 12 μm post-lens [9, 11, 12, 14, 15]. A tear film that is stable between blinks is widely agreed to be a necessity for comfort during contact lens wear [16, 17], but the compartmentalization of the tear film into two thinner segments by the lens can result in a destabilization of this important thin film over the lens surface [18, 19] and increased ocular dryness and discomfort [20].

Several studies have documented this link between an unstable pre-lens tear film and contact lens-related discomfort, dryness, and dissatisfaction. Vidal-Rohr et al., for example, found that at 1 week and 1 month post-dispensing of soft contact lenses with and without a surface coating to improve lubricity, the coated lens had better reported comfort, and also had longer pre-lens tear breakup time measured non-invasively [16]. Rohit et al. found a significant relationship between ocular comfort and lens surface drying time measured non-invasively in 40 habitual soft contact lens wearers (p = 0.003), although the model did not explain a large proportion of the variability (r = 0.21) [17]. Nichols et al. found faster pre-lens thinning time to be significantly related to self-reported dry eye symptoms in 360 contact lens wearers [21]. From numerous studies employing different measures of pre-lens tear film stability, and assessing different subjective outcomes, it has become widely accepted that a primary factor in contact lens discomfort, dryness and dissatisfaction is an unstable pre-lens tear film [20].

A number of studies have also found significant associations of pre-corneal (no contact lens) tear film stability with subsequent contact lens wearing outcomes. Best et al. found that shorter pre-corneal non-invasive tear breakup time (NITBUT) and fluorescein tear breakup time (FTBUT) were both significantly related to CL dropout within 6 months [22]. Baseline pre-corneal NITBUT in particular averaged approximately 5 sec faster among those who dropped out within 6 months, compared with those who were still successfully wearing lenses at 6 months. Predictive performance was moderate, however, with a sensitivity of 63% and a specificity of 76%, which are broadly in line with published estimates for other outcomes related to symptomatology [23, 24]. Glasson et al. found that tolerant soft contact lens wearers had significantly longer pre-corneal NITBUT than did intolerant wearers [25]. Yeh et al. also found significant relationships between pre-corneal NITBUT and FTBUT and visual analog scale (0–100) ratings of the severity and frequency of contact lens-related dryness [26].

As pre-corneal tear film stability is likely related to subsequent comfort during contact lens wear, it could be hypothesized that pre-corneal tear film instability leads to an even more unstable pre-lens tear film during contact lens wear, thus increasing the likelihood of contact lens discomfort, dissatisfaction and dropout [27, 28]. Wong et al. found a significant relationship between baseline pre-corneal and subsequent pre-lens NITBUT, with pre-lens breakup averaging 2.6 sec faster [29]. Kopf et al. found measures of tear film surface quality measured non-invasively by videokeratoscopy to significantly worsen when a soft contact lens was placed on the eye [19].

If it were possible to measure pre-corneal TBUT non-invasively and predict what the pre-lens TBUT will likely be without first having to fit a contact lens, it could significantly aid the clinician by identifying those patients who are unlikely to have sufficient pre-lens tear film stability for successful, symptom-free lens wear. This has not been widely studied, and what results there are in the literature are equivocal [29–32]. In this study, we examine data from a multi-study database of soft contact lens wearers to address the following questions: (a) is the pre-corneal NITBUT significantly related to the pre-lens NITBUT observed 10 min post-insertion?; (b) does the same relationship hold 2–6 hrs post-insertion?; (c) if significant relationships exist, to what extent can the pre-corneal NITBUT be used predictively to identify prospective contact lens wearers who are more likely to remain free of symptoms of discomfort and dryness?

## Methods

### Ethics statement

All subjects participating in this research were oriented as to the goals, procedures, and potential risks and benefits of the studies, after which written informed consent was obtained from all subjects. All studies contributing to the database used in this analysis were approved by the University of California, Berkeley Committee for the Protection of Human Subjects This study adhered to the tenets of the Declaration of Helsinki, and was HIPAA compliant with respect to data safety and subject anonymity.

### Study data and procedures

The data for this analysis were selected from a multi-study database of soft contact lens wear studies conducted at the University of California, Berkeley Clinical Research Center (CRC). All studies eligible for selection employed identical inclusion/exclusion criteria, including being free of ocular infection or inflammation, having no history of prior ocular surgery, and not taking any ophthalmic medications or systemic medications with effects on the ocular surface or tear film. All subjects were experienced, current soft contact lens wearers who had been wearing lenses for at least 8 hrs per day, at least 5 days per week, for at least the prior month. The protocols for all eligible studies included a pre-corneal NITBUT measurement after at least 24 hrs of discontinued lens wear, and a pre-lens NITBUT measurement at either ~10 min post-insertion (i.e., after lens settling), between 2 and 6 hrs post-insertion, or both. These time frames were chosen to reflect a range of recommended examination times of newly-fitted CL wearers for lens fit, performance, safety, and symptomatology [33–35]. In all eligible studies, NITBUT was measured 3 times using a corneal topographer (Medmont E300, Medmont International PTY LTD, Australia) and averaged. The automated timing feature was not used due to the inadequate video frame rate, and more accurate NITBUT measurement at a finer resolution can be achieved by a well-trained observer using a digital stopwatch. Measurements were taken alternating between eyes, and the time between measurements was < 1 min, during which the subject blinked normally, followed by 3 full deliberate blinks prior to the next measurement. Breakup was considered to have occurred at the first sign of distortion in the reflected image of the mires, and all observers were trained and calibrated to the same criteria for identifying the first moment of distortion.

### Statistical methods

After a thorough exploratory analysis, a linear mixed effects modeling approach was employed to estimate the pre-lens NITBUT at 10 min and 2–6 hrs post-insertion of either hydrogel or silicone hydrogel soft contact lenses. The pre-lens NITBUT was modeled as a function of pre-corneal NITBUT measured prior to lens insertion, and adjustments for covariates including age, gender, ethnicity, contact lens material and investigator were examined. Eyes were considered to vary randomly within subjects, and a compound symmetric covariance structure was assumed. Models were compared by F-test p-value, residual and other diagnostic plots, the log-Likelihood for nested models and Akaike’s Information Criterion for non-nested models. NITBUT was truncated at 30 sec and square root-transformed to better approximate normality of residuals. The sensitivity and specificity of pre-corneal NITBUT to predict whether pre-lens NITBUT would be below thresholds linked to symptoms were estimated using Receiver Operating Characteristic (ROC) analysis. Previous work by the authors [24] established that symptoms of contact lens-induced dryness were linked to a pre-lens NITBUT of < 2.5 sec, and that severe dryness symptoms were linked to a pre-lens NITBUT of < 2.0 sec. In addition to the full dataset analysis, the analysis was repeated on the subset of data with pre-corneal NITBUT < 10 sec, a long-established threshold commonly used in clinical practice [36, 37], in order to determine its relationship to pre-lens NITBUT and its predictive performance in a group of subjects with clinically questionable tear film stability prior to lens wear.

## Results

### Data selection

Six studies in the CRC soft contact lens study database were determined to meet eligibility criteria for inclusion in this analysis. All studies included measurement of the pre-corneal NITBUT after a minimum of 24 hrs discontinuation of lens wear, and prior to insertion of lenses at the study visit. A single study measured the pre-lens NITBUT at 10 min post-lens insertion only, one study measured pre-lens NITBUT at 2 hrs post-insertion only; the other four studies measured pre-lens NITBUT at both 10 min and 2–6 hrs post-insertion. One hundred thirty-nine (139) subjects contributed a total of 667 records to the database, with fellow eyes included, and some study protocols requiring multiple visits and some subjects participating in multiple studies. For the comparison of pre-corneal NITBUT to pre-lens NITBUT at 10 min post-contact lens insertion, 348 records from five studies met the inclusion criteria. Two hundred sixty-three (263) of these records were included in the sub-group analysis of those with unstable tear films (i.e., pre-corneal NITBUT < 10 sec). For the comparison at 2–6 hrs post-insertion, 647 records from five studies met the inclusion criteria, with 514 included in the unstable tear film sub-group analysis. The data for this analysis were collected between the years 2009 and 2018 by 6 different investigators, each of whom primarily performed data collection for one study.

### Baseline subject characteristics

Subjects ranged in age from 18 to 41 years, with a mean (SD) age of 22.9 (4.2) years. Subjects were 74.2% female and 25.8% male, 48.4% of Asian ethnicity and 51.6% non-Asian. The Asian sub-group was defined as subjects of Chinese, Vietnamese, Japanese, Korean or Pacific Islander descent. The non-Asian ethnic group was defined to consist of Caucasian, Latino, African-American or Indian subjects.

Seventeen different soft contact lens brands were worn for these studies, with 48.4% being of hydrogel material, and 51.6% silicone hydrogel. Asymptomatic contact lens wearers constituted 62.3% of subjects, with 37.7% being symptomatic. A designation of symptomatic was defined as having a score ≥ 4 on the Berkeley Dry Eye Flow Chart (DEFC) [24], which indicates self-reported dryness and discomfort during an average week of lens wear sufficient to interfere with visual activities (e.g., reading, using a computer, wearing contact lenses) either “sometimes” or “usually/always”. This designation was made at baseline (pre-lens insertion), at the first visit for those with repeated visits.

**Fig 1:**
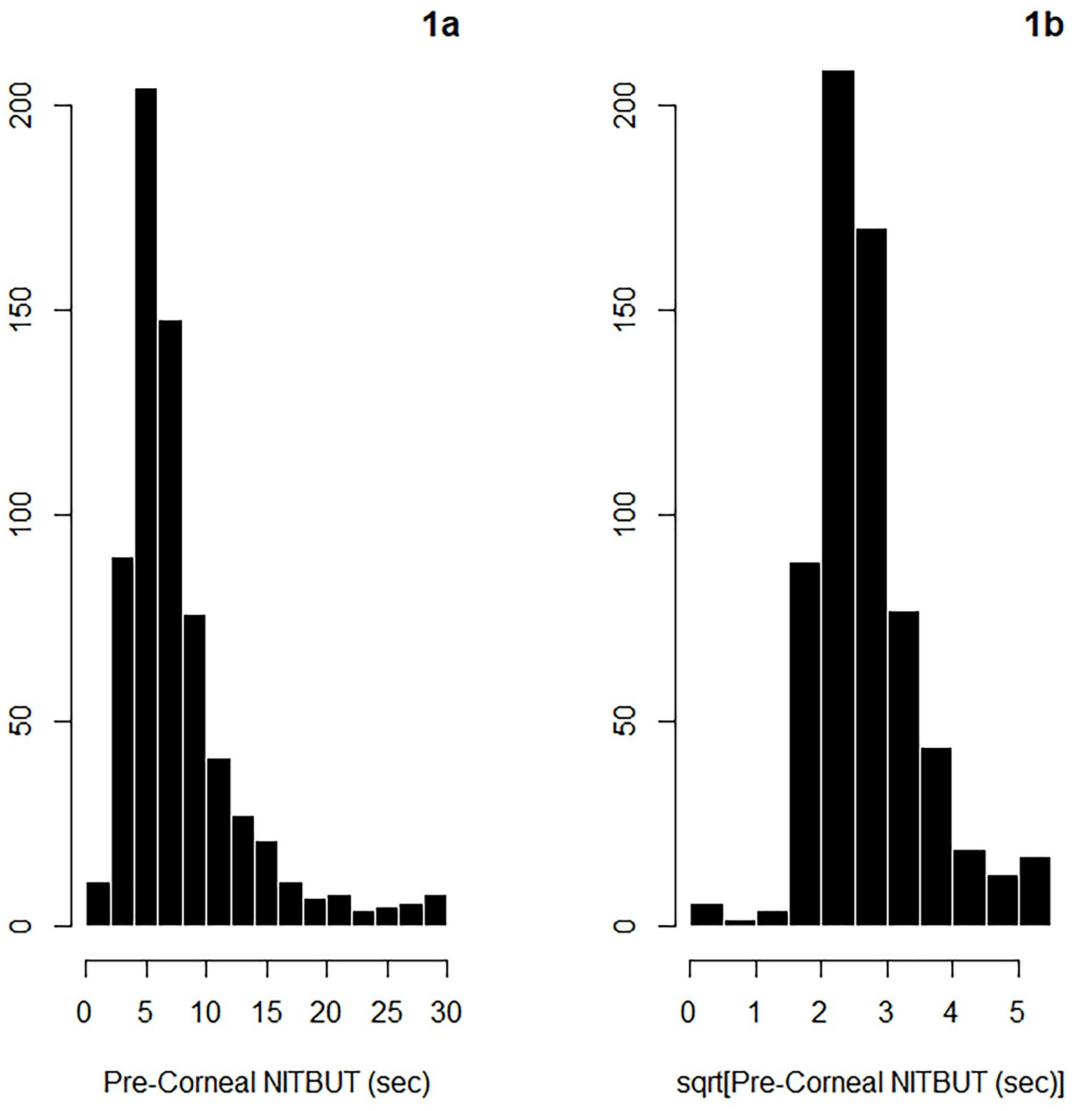
Distributions of raw (1a) and square root-transformed (1b) pre-corneal NITBUT (all data combined, n = 667).

Baseline pre-corneal NITBUT ranged from 0 to the maximum value of 30 sec, with a median of 6.67 sec, and a mean (SD) of 8.48 (5.96) sec. The distribution of baseline pre-corneal NITBUT is depicted in Fig 1a. It is evident from the figure that the distribution of raw NITBUT was right-skewed, therefore we elected to analyze the square root-transformed NITBUT, depicted in Fig 1b.

### Pre-corneal vs. pre-lens NITBUT: 10 min post-insertion

**Fig 2:**
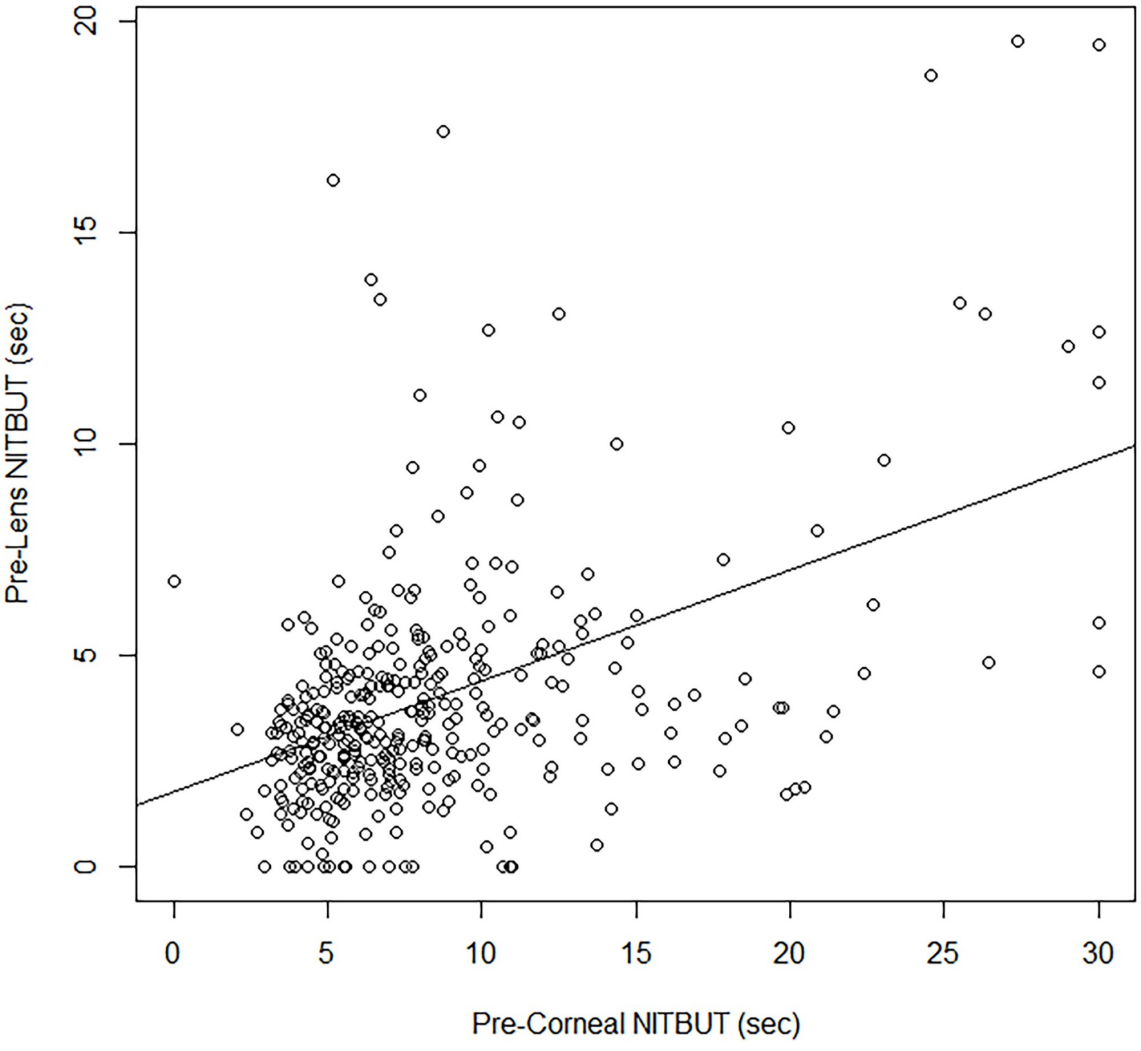
Pre-corneal vs. pre-lens NITBUT at 10 min post-contact lens insertion (n = 348).

The raw data for the comparison of pre-corneal to pre-lens NITBUT at 10 min post-lens insertion are depicted in Fig 2. Modeling the square root of the pre-lens NITBUT as a function of the square root-transformed pre-corneal NITBUT provided the best fit. The pre-lens NITBUT at 10 min post-insertion was significantly related to the pre-corneal NITBUT (p < 0.001) as follows:

$$\sqrt{{PreLens\;NITBUT}_{10min}}\;=\;0.9352\;+\;0.3153\;*\sqrt{PreCorneal\;NITBUT}$$

Adjustment for the different investigators was also significant (p < 0.001), however the difference in the estimated pre-lens NITBUT with and without adjusting for investigators was < 1 sec and thus clinically negligible. Table 1 shows model-estimated pre-lens NITBUT for various values of the pre-corneal NITBUT. The models confirm that the pre-lens NITBUT at 10 min post-insertion is shorter, on average, than the pre-corneal NITBUT. The models also suggest that the difference between the pre-corneal and pre-lens NITBUT increases the more stable the pre-corneal NITBUT is. Pre-lens NITBUT at 10 min post-insertion was not significantly related to subject age, gender, ethnicity, contact lens material (hydrogel or silicone hydrogel), or to being a symptomatic contact lens wearer (as defined above).

**Table 1:** Model estimates and estimated values of pre-lens NITBUT for various values of the pre-corneal NITBUT.

	Estimated Pre-Lens NITBUT (sec)	
Pre-Corneal NITBUT (sec)	10 min Post-Insertion	2–6 hrs Post-Insertion
**1**	1.56	2.15
**3**	2.19	2.69
**5**	2.69	3.09
**7**	3.13	3.44
**10**	3.73	3.91
**15**	4.65	4.60

**Fig 3:**
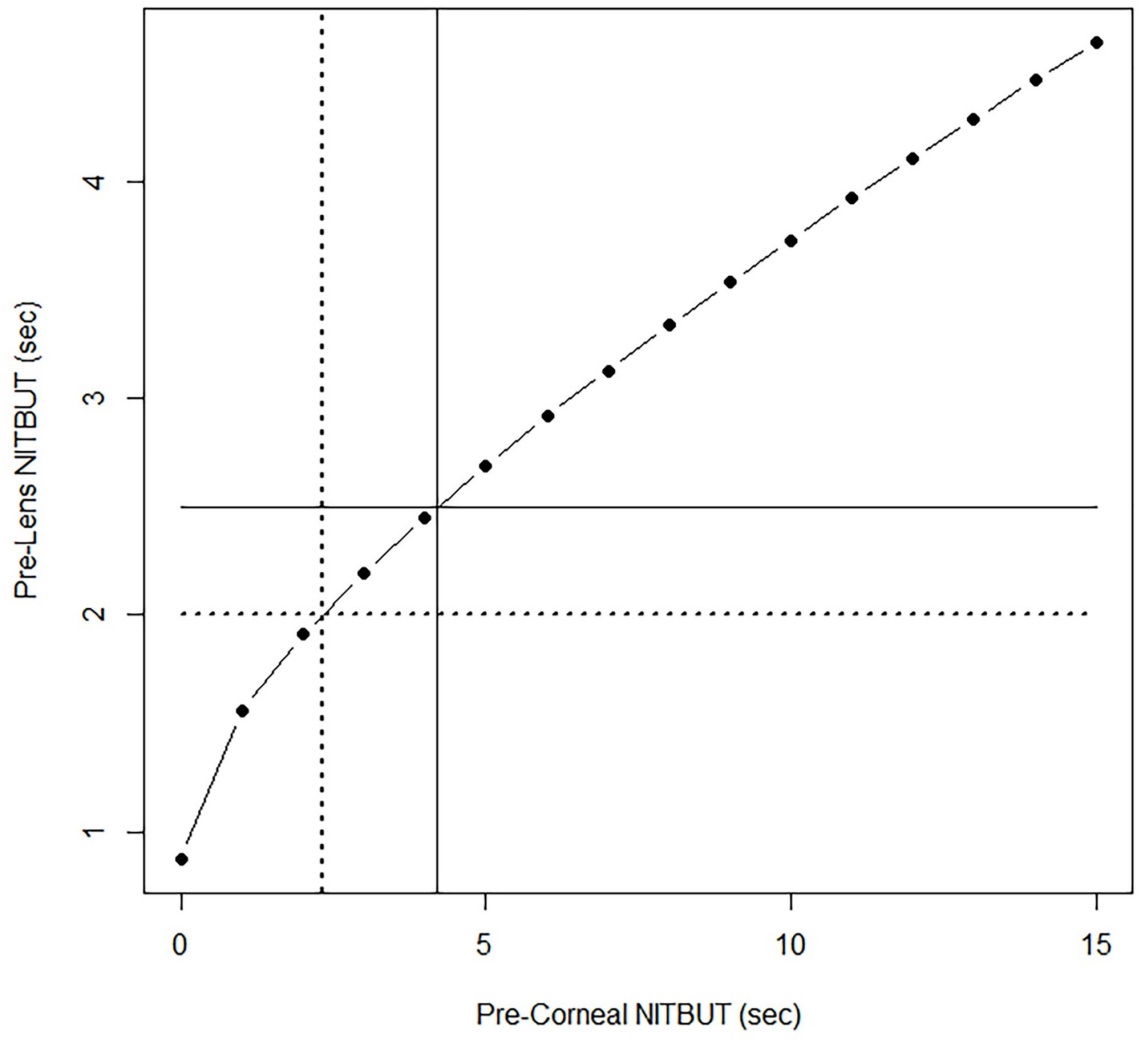
Model estimates for pre-lens NITBUT at 10 min post-contact lens insertion. Crosshairs highlight the pre-corneal NITBUT at dryness symptom-associated thresholds of pre-lens NITBUT (< 2.5 sec for any symptoms, < 2.0 sec for severe symptoms). For these pre-lens thresholds, the associated pre-corneal values are 4.20 sec and 2.31 sec, respectively.

As stated above, contact lens-related dryness symptoms have been linked to a pre-lens NITBUT of < 2.5 sec, and for severe symptoms < 2.0 sec. According to our model estimates, the pre-corneal NITBUT corresponding to a pre-lens NITBUT at 10 min post-insertion of < 2.5 sec is approximately 4.20 sec; the pre-corneal NITBUT corresponding to a < 2.0 sec pre-lens NITBUT at 10 min post-insertion is approximately 2.31 sec (Fig 3). In terms of the actual predictive performance of the pre-corneal NITBUT, we employed ROC analysis to estimate the sensitivity, specificity and optimum threshold to predict a pre-lens NITBUT at 10 min post-insertion of < 2.5 sec and < 2.0 sec. Although the pre-corneal NITBUT was significantly related to the pre-lens NITBUT in the associative model, sensitivity and specificity were poor, at approximately 50% and 57%, respectively for a symptom-associated threshold of 2.5 sec, and approximately 54% and 60%, respectively for a threshold of 2.0 sec (Table 2). Fig 2 clearly shows that, while on average the pre-lens NITBUT rises in direct accordance with the average pre-corneal NITBUT, for any given pre-corneal value, a range of pre-lens values spanning several seconds can be observed, making for poor performance in terms of direct prediction.

**Table 2:** Sensitivity and specificity of pre-corneal NITBUT (sec) for predicting subsequent pre-lens NITBUT at 10 min post-insertion below thresholds associated with any dryness symptoms (2.5 sec) and severe dryness symptoms (2.0 sec).

NITBUT at 10 min Post-Insertion					
	Sensitivity [95% CI]		Specificity [95% CI]		Threshold: Pre-Corneal
**All Subjects**					
** Pre-Lens < 2.5s**	50.0	[40.1, 59.9]	57.2	[53.1, 61.2]	5.86s
** Pre-Lens < 2.0s**	53.8	[41.7, 66.0]	60.2	[56.3, 64.1]	5.59s
**Sub-Group**					
** Pre-Lens < 2.5s**	60.5	[49.8, 71.1]	60.4	[53.3, 67.5]	5.86s
** Pre-Lens < 2.0s**	64.8	[52.1, 77.6]	63.6	[57.1, 70.2]	5.63s

The sub-group consists of subjects with pre-corneal NITBUT < 10 sec. Sensitivity and specificity are given as percentages (n = 263). CI = confidence interval.

In a sub-group analysis, we investigated whether the same relationships would hold amongst those subjects presenting with pre-corneal NITBUT < 10 sec, as those would represent patients with questionable-to-poor tear film stability prior to contact lens fitting. The relationship was significant in the sub-group (p < 0.001) with a similar slope to that for the full group (0.317 and 0.315, respectively). The pre-corneal NITBUT corresponding to 2.5 sec pre-lens NITBUT was 3.86 sec, and corresponding to 2.0 sec pre-lens NITBUT was 2.07 sec. Predictive performance of pre-corneal NITBUT for pre-lens NITBUT at 10 min post-insertion was moderate-to-poor for this sub-group with possible tear film instability. Sensitivity and specificity for predicting pre-lens NITBUT < 2.5 sec were approximately 61% and 60%, respectively, and for pre-lens NITBUT < 2.0 sec were approximately 65% and 64%, respectively (Table 2).

### Pre-corneal vs. pre-lens NITBUT: 2–6 hrs post-insertion

**Fig 4:**
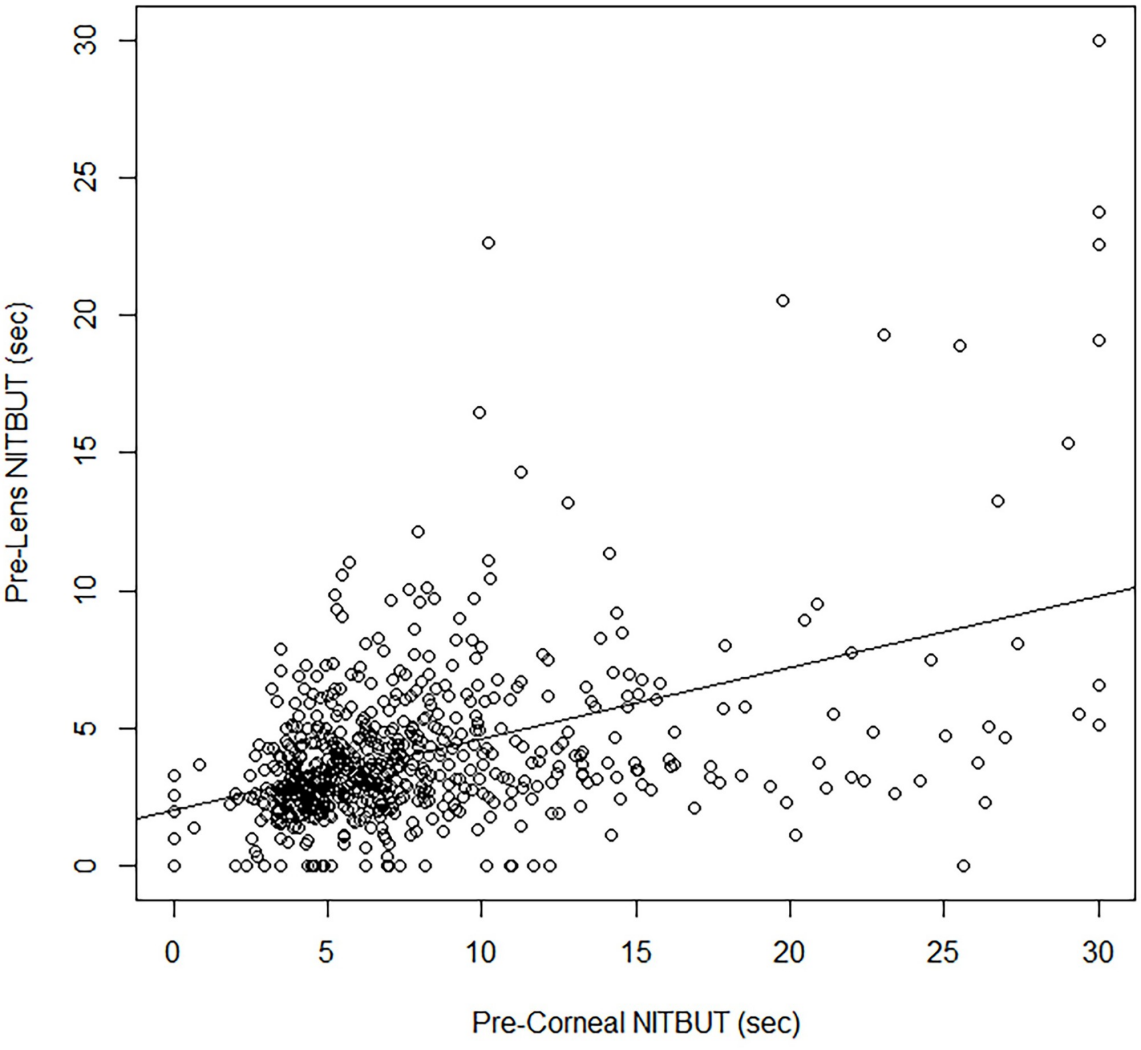
Pre-corneal vs. pre-lens NITBUT at 2–6 hrs post-contact lens insertion (n = 647).

The raw data for the comparison of pre-corneal to pre-lens NITBUT at 2–6 hrs post-lens insertion are depicted in Fig 4. The pre-lens NITBUT at 2–6 hrs post-insertion was significantly related to the pre-corneal NITBUT (p < 0.001) as follows:

$$\sqrt{{PreLens\;NITBUT}_{26\text{hrs}}}\;=\;1.2320\;+\;0.2355\;*\sqrt{PreCorneal\;NITBUT}$$

**Fig 5:**
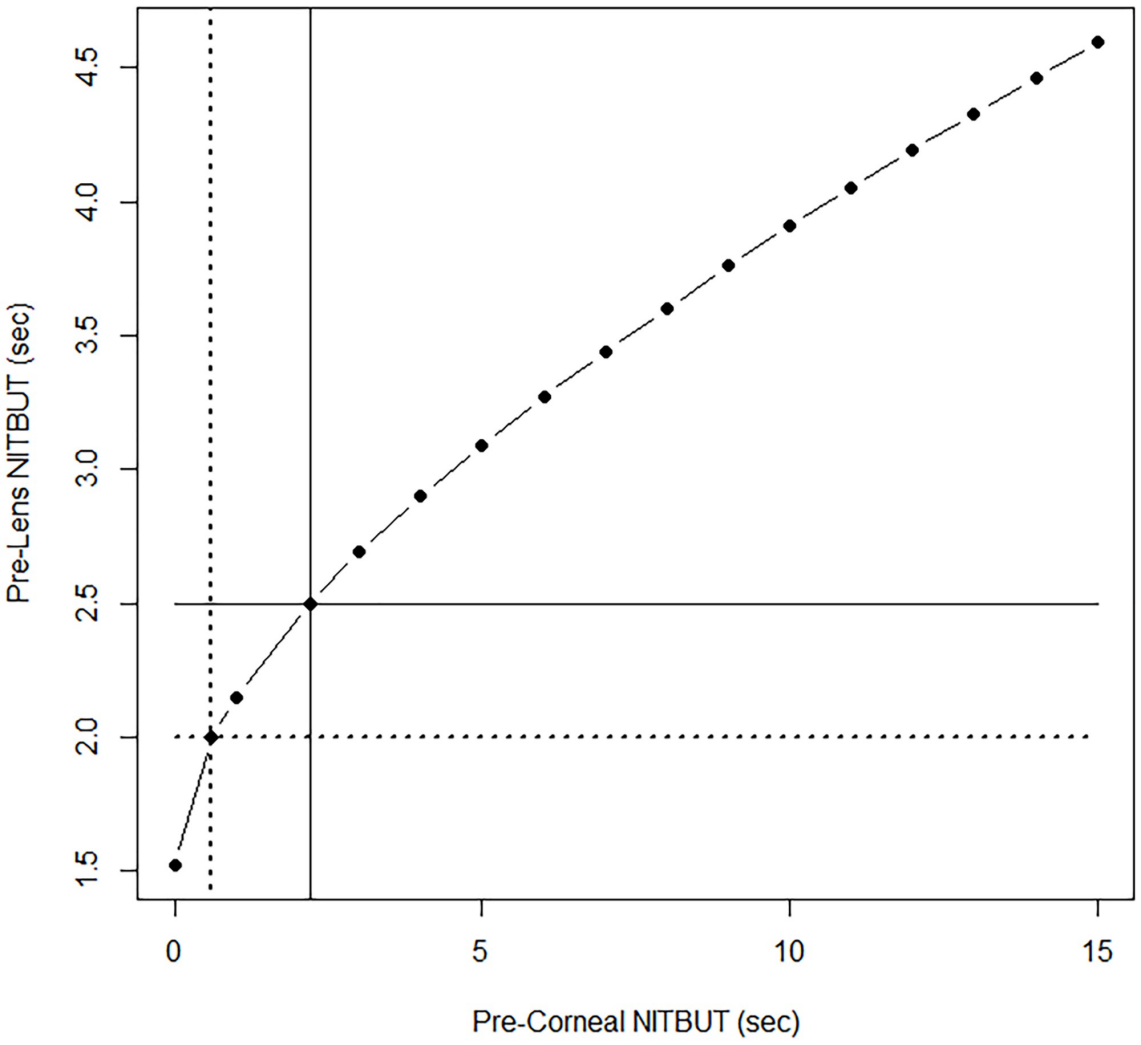
Model estimates for pre-lens NITBUT at 2–6 hrs post-contact lens insertion. Crosshairs highlight the pre-corneal NITBUT at dryness symptom-associated thresholds of pre-lens NITBUT (< 2.5 sec for any symptoms, < 2.0 sec for severe symptoms). For these pre-lens thresholds, the associated pre-corneal values are 2.20 sec and 0.60 sec, respectively.

Pre-lens NITBUT at 2–6 hrs post-insertion was not significantly related to subject age, gender, ethnicity, contact lens material, investigator, or to being a symptomatic contact lens wearer. According to model estimates, a pre-corneal NITBUT of approximately 2.20 sec corresponds to a pre-lens NITBUT of < 2.5 sec at 2–6 hrs post-insertion; a pre-corneal NITBUT of approximately 0.60 sec corresponds to a < 2.0 sec pre-lens NITBUT at 2–6 hrs post-insertion (Fig 5). As with 10 min post-insertion, the sensitivity of pre-corneal NITBUT was poor, at approximately 52% and 53%, respectively, for symptom-associated thresholds of 2.5 sec and 2.0 sec. The specificity, however, was improved at 2–6 hrs post-insertion, at approximately 72% and 69%, respectively for thresholds of 2.5 sec and 2.0 sec (Table 3).

**Table 3:** Sensitivity and specificity of pre-corneal NITBUT for predicting subsequent pre-lens NITBUT at 2–6 hrs post-insertion below thresholds associated with any dryness symptoms (2.5 sec) and severe dryness symptoms (2.0 sec).

NITBUT at 2–6 hrs Post-Insertion					
	Sensitivity [95% CI]		Specificity [95% CI]		Threshold: Pre-Corneal
**All Subjects**					
** Pre-Lens < 2.5s**	52.2	[44.4, 60.0]	72.2	[68.3, 76.0]	5.16
** Pre-Lens < 2.0s**	52.8	[42.4, 63.2]	69.4	[65.6, 73.1]	5.16
**Sub-Group**					
** Pre-Lens < 2.5s**	56.2	[47.9, 64.5]	68.4	[63.7, 73.1]	4.93
** Pre-Lens < 2.0s**	57.1	[46.1, 68.2]	65.2	[60.8, 69.7]	4.93

The sub-group consists of subjects with pre-corneal NITBUT < 10 sec. Sensitivity and specificity are given as percentages (n = 514). CI = Confidence Interval.

In the sub-group analysis of those subjects presenting with pre-corneal NITBUT < 10 sec, the relationship between pre-corneal NITBUT and pre-lens NITBUT at 2–6 hrs post-insertion remained significant (p < 0.001) with a similar slope compared with analyzing the full group (0.197 and 0.236, respectively). The pre-corneal NITBUT corresponding to 2.5 sec pre-lens NITBUT was 1.41 sec, and corresponding to 2.0 sec pre-lens NITBUT was 0.12 sec. Predictive performance of pre-corneal NITBUT for pre-lens NITBUT at 2–6 hrs post-insertion was similarly modest for this sub-group with possible tear film instability. Sensitivity and specificity for predicting pre-lens NITBUT at 2–6 hrs post-insertion < 2.5 sec were approximately 56% and 68%, respectively, and for pre-lens NITBUT < 2.0 sec were approximately 57% and 65%, respectively (Table 3).

## Discussion

In this study, we examined both the associative and the predictive relationships of pre-corneal (no lens wear) tear film stability to pre-lens tear film stability with soft contact lenses *in situ*. While there is a direct and significant associative relationship between pre-corneal and pre-lens tear breakup times, the predictive performance of pre-corneal NITBUT for subsequent tear film stability after inserting a contact lens is not sufficiently strong to be relied upon clinically. We found that over the range of pre-corneal tear breakup times observed, any given pre-corneal value is associated with a range of pre-lens times spanning several seconds. This is also reflected in the moderate-to-poor sensitivities and specificities of pre-corneal NITBUT in predicting whether a potential lens wearer will be above or below symptom-associated pre-lens NITBUT thresholds.

There are several reasons that a given pre-corneal NITBUT cannot accurately predict later pre-lens NITBUT consistently for individuals in this group of contact lens wearers. First is the group itself: our study sample contained a wide range of symptomatic and asymptomatic subjects with pre-corneal and pre-lens tear film stability spanning the full measurement range; what is consistent across this group is that they were all 41 years of age or younger, full-time contact lens wearers in spite of any tear film instability or symptoms of dryness or discomfort. Although NITBUT was measured 3 times and averaged in an effort to mitigate within-subject variability, it is possible that some individual NITBUT measurements, given the known inter-day variability in tear film stability, were values that would not be typical for those subjects, with normal day-to-day tear film stability sufficient to maintain contact lens wear. It could be that a study designed with a larger number of repeated measures per subject (i.e., to get a better picture of each subject’s typical day-to-day values), or in a different study population that exhibited more stable, less variable breakup times, or who were contact lens neophytes [38], would find a closer predictive relationship between pre-corneal and pre-lens NITBUT.

A second broad reason for poor predictive performance are the lability and exposure sensitivities of tear breakup times [39–41]. Individuals often exhibit clinically significant differences in breakup times from one day to the next, at different times of day, after different visual tasks (e.g., distance viewing with normal blinking vs. prolonged near work with reduced blinking), and after various environmental exposures (e.g., eye makeup or facial skin care products, excessive wind or dust, low humidity, swimming). The fact that normal tear film stability naturally varies a great deal [42], coupled with its sensitivity to the multiple, often overlapping exposures to which most people are subject in day-to-day life, together result in a pre-corneal measurement that is consistently associated with pre-lens measurements on average in a large group, but that is subject to too much inherent variability to be of individual predictive value to the clinician. A carefully conducted prospective study employing a controlled environment chamber would likely result in better predictive performance, however this would be unlikely to be of much value to the clinician for the reasons that (a) few if any clinical practices are equipped with such expensive, specialized equipment, and (b) predictive results obtained under such controlled conditions would bear little resemblance to conditions obtaining in the real world.

Another contributor to the lack of good predictive performance is the method of NITBUT itself to assess tear film stability. The sensitivity of NITBUT to detect tear film instability depends on the number, size, and spacing of the reflected mires. Tear film instability can occur without being captured by the reflected mires, making the measurements potentially less sensitive to the initiation of instability due to the potential lack of detection. From the larger perspective of comparing results across the literature, this also means that results from different NITBUT instruments with different mire sizes and densities may vary from one to another in terms of diagnostic performance. Lastly, one may postulate that the poor performance of pre-corneal NITBUT for predicting pre-lens NITBUT is due to different physical phenomena that determine how pre-corneal and pre-lens tear films deposit over the corneal and the contact lens surfaces, respectively, as different radii of tear menisci may be formed [43] before and during lens wear.

It is interesting to note that in our models of pre-lens tear film stability, the longer the pre-corneal NITBUT the bigger the difference between the pre-corneal NITBUT and the estimated pre-lens NITBUT. For example, at 10 min post-insertion a pre-corneal NITBUT of 5 sec is associated with a pre-lens NITBUT of 2.69 sec–a difference of 2.31 sec; for a pre-corneal NITBUT of 15 sec, the estimated pre-lens NITBUT is 4.65 sec–a difference of 10.35 sec. Although only suggestive from these data, it is possible that above a certain threshold, no additional extensions of pre-corneal tear film stability will result in additional stability once a lens is in place. In other words, there may be an upper limit to the length of time a tear film can remain stable on the surface of a soft contact lens. This could have implications for both contact lens manufacturers and clinicians in contact lens practice, and warrants further investigation with a properly designed study involving a greater sample size and a variety of lens materials.

Models from the current study estimate that at 10 min post-lens insertion, a pre-corneal NITBUT of 4.2 sec corresponds to a pre-lens NITBUT of 2.5 sec (the threshold for dry eye symptoms), and a pre-corneal NITBUT of 2.3 sec corresponds to a pre-lens NITBUT of 2.0 sec (the threshold for severe dry eye symptoms). This is in agreement with other studies that have shown that on average, the thinner, pre-lens segment of the compartmentalized tear film with lenses *in situ* breaks up faster than does the whole tear film without lenses [42, 44]. After 2–6 hrs post-insertion, however, models estimate that a pre-corneal NITBUT of 2.2 sec corresponds to a 2.5 sec pre-lens NITBUT, and a pre-corneal NITBUT of 0.6 sec corresponds to a 2.0 sec pre-lens NITBUT. This counterintuitive result, in which the pre-corneal tear film is estimated to have a shorter breakup time than the pre-lens tear film in the first few seconds after eye opening, may be due in part to the paucity of data below 3 sec (n = 26, or ~4%). Above 3 sec, in which our models estimate the pre-lens NITBUT to be shorter than the pre-corneal as expected (see Table 1), is where the bulk of the data (n = 621, or ~96%) lie. The fitted line below 3 sec gives these estimates not because of the very few data points in that range, but because of the vast majority of the data above 3 sec that determine the slope. Additional data in the very short and long NITBUT ranges would likely improve the models fits.

A further source of variability in pre-lens NITBUT readings that affects the fit of the models and the reliability of predictions is the possibility of undetected, low-level reflex tearing. Although readings with lenses on that had obviously visible reflex tearing at the study visits were rejected and measurements redone, there was possibly some lower-level reflex tearing in some subjects that was not detected at the time and that contributed to apparently longer NITBUT with lenses on than without lenses. Although it cannot be determined from video evidence, if this is indeed the case (n.b., as might be expected to occur more often with lenses on due to mechanical interaction with the cornea), the predictive ability of pre-corneal NITBUT might be improved with more detailed assessment of possible reflex tearing with lenses (e.g., measuring small changes in tear meniscus parameters). Beyond some cases of low-level reflex tearing, NITBUT times vary a great deal even within individuals, often by many seconds from one measurement to the next. It is also possible that some subjects happened to have relatively fast pre-corneal breakup on that particular measurement, and then transitioned temporarily to a more stable state when pre-lens NITBUT was being measured, simply due to natural variability in tear film stability. On an individual basis, the exact correspondence of pre-corneal to pre-lens tear film stability is difficult to ascertain; it is only on average for a large group that there are sufficient data to overcome the inherent variability of NITBUT measurements and observe the significant association between pre-corneal and pre-lens NITBUT.

Taken as a whole, these results suggest that factors other than native pre-corneal tear film stability can impact pre-lens tear film stability for many people. Young and Efron found, for example, that pre-lens NITBUT times depended on the water content of hydrogel lens materials [44]. Bruce et al. demonstrated that having an Etafilcon-A lens on the eye resulted in different tear breakup locations than on the cornea without lenses [45]. Lens materials can have effects on the composition, and thus the stability characteristics, of the pre-lens tear film. Mann et al. suggest that the pre-lens portion of the compartmentalized tear film–particularly the lipoidal components–is subject to increased autoxidative degeneration, and that the lens material itself can deplete the tear film by absorbing or reducing certain tear components [46]. In our laboratory setting, factors such as temperature, humidity, wind speed, and environmental exposures are controlled (e.g., airborne particulates are filtered, eye makeup is prohibited during study), but are likely to result in additional uncontrolled effects on the pre-lens tear film in the real-world clinical setting. In summary, it is clear that while pre-corneal and pre-lens tear film stability are directly related and can provide useful data for investigating research questions statistically, in the clinical setting a patient having good individual pre-corneal tear film stability at a single visit is not a good predictor that that individual will maintain a stable tear film once fit with a contact lens.
